# Lower Patient Height and Weight Are Predisposing Factors for Complex Radial Arterial Catheterization

**DOI:** 10.3390/jcm12062225

**Published:** 2023-03-13

**Authors:** Kristine Huber, Jan Menzenbach, Markus Velten, Se-Chan Kim, Tobias Hilbert

**Affiliations:** 1 Department of Anesthesiology and Intensive Care Medicine, University Hospital Bonn, Venusberg-Campus 1, 53127 Bonn, Germany; 2 Department of Anesthesiology, Perioperative Care and Pain Medicine, RKH Hospital gGmbH, Kurt-Lindemann-Weg 10, 71706 Markgröningen, Germany

**Keywords:** ultrasound, sonography, arterial catheterization, invasive blood pressure monitoring

## Abstract

**Background:** Radial artery (RA) catheterization for invasive blood pressure monitoring is often performed via palpation, and an ultrasound is used secondarily only in case of multiple unsuccessful attempts. Although more elaborate, it has been shown that primary ultrasound-guided catheterization may be advantageous compared with palpation. The aim of this study was to identify factors associated with difficult RA catheterization. **Methods:** Left RA ultrasound assessments were performed in patients with indicated invasive blood pressure monitoring the day before surgery. RA catheterization was performed by personnel blinded to the ultrasound results. Based on the number of attempts needed for successful catheter placement, the cohort was divided into uncomplicated (group 1) and difficult (more than one attempt, group 2) catheterization cases. Cases subjected to primary ultrasound were excluded from the analysis. **Results:** Body weight, height and surface area (BSA) of patients in group 2 (*n* = 16) were significantly lower than those of patients in group 1 (*n* = 25), and internal RA diameters were significantly smaller in group 2 patients. In the whole cohort, BSA was significantly associated with distal and proximal internal RA diameters. In contrast, no differences were observed in the skin-to-artery distance, the longitudinal axis deviation (kinking) or blood flow velocity. Median time to successful catheterization was 77 (47–179) s. Prolonged time needed for cannulation was significantly associated with lower body weight, BMI and BSA, and with reduced distal and proximal internal RA diameter. **Conclusions:** RA catheterization performed through pulse palpation may be difficult, especially in adult patients with lower body weight and height, due to reduced arterial diameters. Initial use of ultrasound in these patients may reduce first-attempt failure, preventing procedural delays and patient discomfort.

## 1. Background

Blood pressure monitoring is standard during anesthesia care. While non-invasive assessment based on the Riva-Rocci method is usually assumed to be sufficient for patients presenting with moderate comorbidities and for minor invasive interventions, continuous invasive (“bloody”) intra-arterial blood pressure monitoring is recommended for more invasive procedures or for critically ill patients [1]. This requires the insertion of an arterial intravascular catheter, with the radial artery being commonly accessed [2]. Using a fluid-filled pressure conduction line connected to the catheter, beat-to-beat intra-arterial blood pressure can be assessed using a pressure transducer placed outside the patient’s body and then displayed on a monitor. The need for higher and frequent arterial blood gas sampling, e.g., during thoracic surgery involving challenging ventilation and oxygenation, is a further indication of the requirement for arterial cannulation [1,3]. Moreover, transradial access has been established as a preferred access for percutaneous coronary catheterization [4].

Radial arterial puncture is usually performed with the index or middle finger of the hand opposite to that palpating the artery. Although it has been shown that primary ultrasound-guided puncture may be advantageous over palpation [5], in clinical practice, vascular sonography followed by secondary ultrasound-guided puncture are utilized only in case of multiple unsuccessful attempts. This results in unwanted delays and may be associated with increased complication rates [5].

Additionally, due to procedural reasons or low availability of ultrasound devices, it is often not possible to perform every arterial catheterization ultrasound-guided. Therefore, the aim of this study was to identify those factors that are associated with difficult puncture or catheterization and that therefore make primary vascular sonography appear reasonable. Surgical patients with a need for invasive blood pressure monitoring were evaluated using vascular ultrasounds the day before their planned catheterization, and the results of the assessments were correlated with punctures performed by an operator blinded to those results.

## 2. Methods

All analyses were performed in accordance with the Declaration of Helsinki. The local ethics committee (University Hospital Bonn, Germany) considered the study to be compliant with the applicable professional codes and regulations and therefore approved the study protocol (Ethics Committee of the University Hospital Bonn, Germany; protocol number 261/19; date of approval: 28 August 2019). Written informed consent was obtained from all patients before ultrasound examination and catheterization. Elective orthopedic and cardiac surgery patients aged >18 years with indicated invasive blood pressure monitoring were included. Exclusion criteria were emergency surgery and refusal to provide written informed consent. 

Radial artery (RA) ultrasound assessment was performed the day before surgery. As per internal standard, arterial catheterization is usually performed on the left side; therefore, left RA ultrasound characteristics were assessed. All assessments were performed by one trained person experienced in anesthesiologic vascular ultrasound using a linear array hockey stick probe (L15-7io) on a Philips CX50 ultrasound machine (Philips GmbH, Hamburg, Germany). The following parameters were obtained:

RA diameter at the level of and 5 cm proximal to the styloid process.
Longitudinal axis deviation over a distance from the level of styloid process to 5 cm proximal to it (kinking of the artery).
Skin-to-artery distance at the level of and 5 cm proximal to the styloid process.
Maximum blood flow velocity (Vmax).
Presence of stenoses and plaques.
RA diameter and axis deviation measured longitudinally (long axis, LA) and cross-sectionally (short axis, SA).

Catheterization of the RA was performed and observed the day following ultrasound examination. The puncture itself was performed either without or primarily or secondarily using ultrasound guidance at the discretion of the operator who was not part of the study personnel and was therefore blinded to the results of the ultrasound assessment. All catheterizations in both groups (see below) were performed uniformly by the same three operators, all of whom were board-certified anesthesiologists with years of experience in performing arterial and venous vascular punctures. In all cases, punctures were performed at the level of the proximal radial artery, approximately 2 cm proximal to the radial styloid (to be differentiated from the recently described distal radial access, which is performed at the level of the snuffbox [6,7]). All catheterizations performed primarily using ultrasounds were excluded from subsequent analyses. Based on the number of attempts needed to successfully place the catheter, the cohort was divided into uncomplicated and difficult (more than one attempt) catheterizations (group 1 or 2, respectively). The following details were recorded:

Number of attempts needed for successful catheter placement.
Time needed for successful catheter placement.
Secondary use of ultrasound.
RA punctures themselves were performed by the same three trained anesthetists according to local hospital standards. Additional data recorded comprised:
Body weight and height.
Heart rate and systolic and diastolic blood pressure during sonography as well as during catheterization.
Pulsatility index obtained from pulse oximetry during catheterization.

Body surface area (BSA) was calculated using the formula by DuBois: BSA (m^2^) = body height^0.725^ (cm) × body weight^0.425^ (kg) × 0.007184.

Statistical analyses and visualizations were performed using MS Excel 2019 (Microsoft Corp., Redmond, CA, USA) and GraphPad PRISM 8 (La Jolla, CA, USA). Data are presented as median values with 25th and 75th percentiles or as absolute numbers with percentage values and were analyzed using Mann–Whitney test and Fisher’s exact test, respectively, and Spearman’s correlation. The alpha level was set to 0.05. All datasets are available from the corresponding author on reasonable request.

## 3. Results

**Figure 1:**
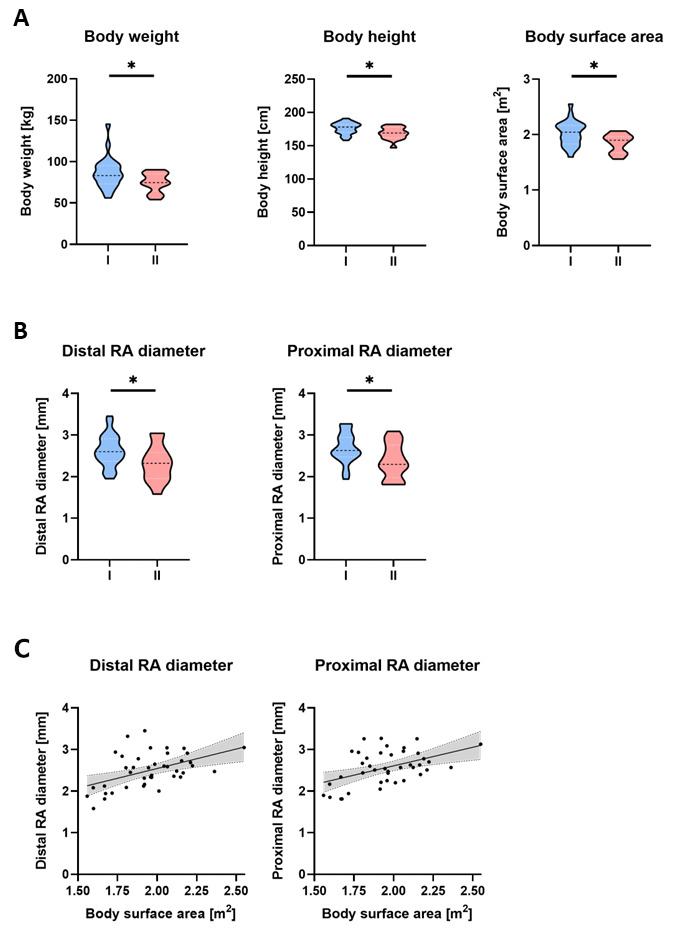
Body metrics and preoperative radial artery ultrasound. Ultrasound assessment of the left radial artery (RA) was performed in all patients the day before surgery using a linear probe. According to the number of attempts needed for successful subsequent RA catheterization, the cohort was divided into un-complicated and difficult (more than one attempt) catheterization (group 1 (*n* = 25) or 2 (*n* = 16), respectively). (**A**) Patients in group 2 (difficult catheterization) had lower body weight, height and surface area than those in group 1. (**B**) In group 2 (difficult catheterization), distal (at the level of the styloid process) and 5 cm proximal internal RA diameter was significantly smaller compared to those in group 1. (**C**) In the whole cohort, body surface area was significantly associated with distal as well as proximal internal RA diameter. Data are visualized as violin diagrams with median and interquartile range (25–75), indicated by the dashed lines. Mann-Whitney test and Spearman correlation were used for analysis. * *p* < 0.05.

In total, ultrasound assessments—conducted the day before surgery—and arterial catheterizations were performed in 47 patients. As the anesthetist performing arterial puncture was blinded to the results of the pre-procedural ultrasound assessment and the approach to cannulation was left at their discretion and solely observed, punctures were performed either without or primarily or secondarily (when catheterization appeared difficult) using ultrasound guidance. Only those procedures involving catheterization via palpation were included in the analysis, while those with primary use of sonography (*n* = 6) were excluded. Difficult cannulation was defined as the need for multiple attempts (more than one) to successfully puncture the artery, and accordingly, the cohort was divided into uncomplicated (group 1, *n* = 25) and difficult (group 2, *n* = 16) catheterization. The study included cardiac surgery (*n* = 36) and orthopedic surgery (*n* = 5) patients. Median body weight in the whole cohort was 82 (73–90) kg, height was 174 (167–181) cm, BMI was 26.8 (23.8–29.2) kg/m^2^ and BSA was 1.96 (1.80–2.12) m^2^. In group 1, median body weight was 83 (73–95) kg, height was 178 (170–182) cm, BMI was 27.7 (24.2–29.1) kg/m^2^ and BSA was 2.04 (1.83–2.17) m^2^. In group 2, median body weight was 75 (65–85) kg, height was 169 (162–178) cm, BMI was 25.9 (22.7–29.3) kg/m^2^ and BSA was 1.90 (1.67–1.96) m^2^. Body weight (*p* = 0.046), height (*p* = 0.011) and BSA (*p* = 0.011) of patients in group 2 were significantly lower than those of patients in group 1 (Figure 1A). Heart rate and blood pressure showed no significant intergroup differences.

Table 1 provides the results of the preoperative ultrasound assessments for the whole cohort as well as for groups 1 and 2. As shown in Figure 1B, the distal and proximal internal radial artery diameters were significantly smaller in group 2 patients (difficult catheterization) compared with group 1 patients (*p* = 0.02). In the whole cohort, Spearman’s correlation analysis showed that BSA was significantly associated with distal (Spearman’s r = 0.48, *p* = 0.001) as well as proximal internal radial artery diameters (Spearman’s r = 0.40, *p* = 0.009). In contrast, no intergroup differences were observed in the depth of the artery beneath the skin, in the longitudinal axis deviation over a distance of 5 cm or in blood flow velocity. Furthermore, plaques and stenoses had no influence on the difficulties encountered during cannulation.

Ultrasound assessment of the left radial artery (RA) was performed in all patients the day before surgery using a linear probe. Based on the number of attempts needed for successful subsequent RA catheterization, the cohort was divided into uncomplicated (group 1, *n* = 25) and difficult (more than one attempt, group 2, *n* = 16) catheterization.

Patients in group 2 (difficult catheterization) had lower body weight, height and surface area compared with patients in group 1.

Distal (at the level of the styloid process) and 5 cm proximal internal RA diameters were significantly smaller in group 2 (difficult catheterization) than in group 1.

Body surface area of the whole cohort was significantly associated with distal as well as proximal internal RA diameters. 

Data are visualized as violin diagrams with the median and interquartile range (25–75), indicated by the dashed lines. The Mann–Whitney test and Spearman’s correlation were used for analysis (*p* < 0.05).

Time needed for successful radial artery (RA) catheterization with lower body weight, height and surface area as well as with smaller distal (at the level of the styloid process) and 5 cm proximal internal RA diameters were measured.

**Figure 2:**
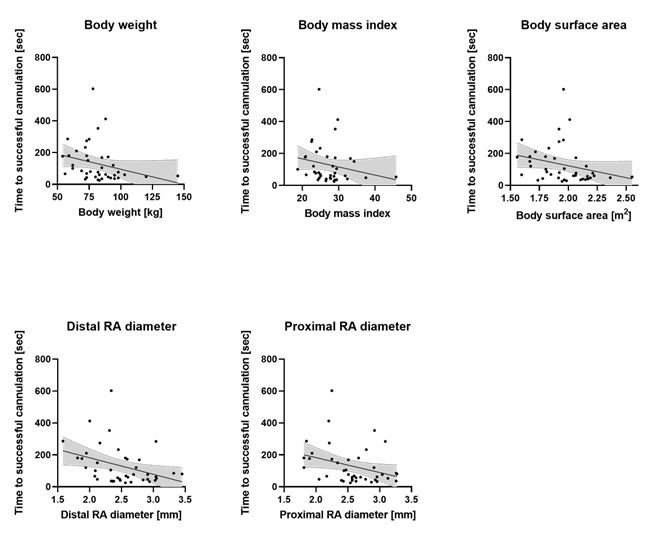
Radial artery catheterization. Time needed for successful radial artery (RA) catheterization with lower body weight, height and surface area as well as with smaller distal (at the level of the styloid process) and 5 cm proximal internal RA diameter. Spearman correlation was used for analysis.

Median time needed for successful catheterization was 77 (47–179) s for the whole cohort. As expected, the need for multiple attempts significantly prolonged the time needed for catheterization in group 2 (181 (155–286) s) compared with group 1 (53 (38–77) s, *p* < 0.0001). Furthermore, difficult arterial puncture with a need for multiple attempts significantly increased secondary ultrasound use in group 2 (50%) compared with group 1 (0%, *p* = 0.0001). Spearman’s correlation analysis of the whole cohort showed that the prolonged time needed for cannulation was significantly associated with lower body weight (Spearman’s r = −0.41, *p* = 0.008), BMI (Spearman’s r = −0.31, *p* = 0.049) and BSA (Spearman’s r = −0.40, *p* = 0.009), and with reduced distal (Spearman’s r = −0.38, *p* = 0.014) and proximal (Spearman’s r = −0.33, *p* = 0.033) internal radial artery diameters (Figure 2). No intergroup differences were observed in heart rate, systolic and diastolic blood pressure or pulse perfusion index during catheterization, or in the secondary intraoperative failure of invasive blood pressure monitoring. Results of the catheterization observations in the whole cohort as well as in group 1 and group 2 are given in Table 2.

## 4. Discussion

Ultrasound guidance can be beneficial during radial artery catheterization. However, the factors that may impede puncture and therefore make primary use of sonography advantageous are still elusive. We demonstrated that difficult radial artery cannulation for invasive blood pressure monitoring resulting in the need for multiple attempts significantly prolonged the procedural processes. This was associated with reduced proximal as well as distal radial artery diameters and was seen particularly in patients with lower body weight and height. Primary ultrasound use may be advantageous in these patients.

Radial artery catheterization is a common invasive procedure in anesthesia care as it allows for real-time continuous blood pressure monitoring in critically ill patients as well as during more invasive surgical procedures, and for repeated blood sampling, e.g., blood gas analyses [1,2,3]. Usually, puncture and catheterization of the radial artery are performed using anatomic landmarks and pulse palpation. However, although widely established in daily practice, this technique may fail, especially under circumstances such as arterial hypotension or in small infants [1,2]. The resulting multiple attempts lead to delays in procedural processes and may induce secondary complications such as hematoma or temporary—or even permanent—vascular occlusion with subsequent distal necroses [1,2,8,9]. Ultrasound guidance for vascular access and regional anesthesia allows for direct visualization of targeted vessels and nerval structures and the puncture process and can provide confirmation of the correct guidewire and catheter positioning, and is therefore established in anesthesia and intensive care medicine and recommended in recent guidelines [10,11,12,13]. However, in contrast to venous access and nerve blockade, radial artery cannulation is still commonly performed via palpation, and sonography is only used when multiple attempts have failed. In addition to delays and complications, this greatly impairs patient comfort. 

In daily practice, operation room workflows may be significantly affected by difficulties encountered while establishing vascular access, greatly impacting cost efficiency [9]. As demonstrated by our results, in case a first attempt performed using palpation fails, the need for further attempts with or without ultrasound obviously delays the whole procedural workflow. A meta-analysis by Gu et al. revealed that radial artery catheterization first-attempt failure was significantly reduced by the use of 2D ultrasound, as were hematoma complications and the mean time needed for successful cannulation [5]. Similar results have been shown for alternative radial access routes such as the distal approach performed at the level of the snuffbox, with ultrasound reducing complications and maximizing technical success, even in small-diameter or pathological arteries [6]. Moreover, the results of the RAUST trial (Radial Artery Access With Ultrasound Trial) demonstrated the advantages of ultrasound use in radial artery cannulation in a randomized multicenter setting [14].

Performing all radial artery catheterization using an ultrasound is possible in centers with high availability of ultrasound machines, but is not possible at our tertiary university medical center. To identify anatomical and physiognomical factors associated with difficult palpational punctures would therefore be of particular interest since this would aid in stratifying patients for primary or secondary ultrasound guidance. Based on our results, reduced distal and proximal radial artery diameters were significantly associated with multiple catheterization attempts and prolonged the time needed for successful catheterization. Since low body weight and height were predisposing factors for reduced arterial diameter, cannulation was difficult in patients with lower body mass index or body surface area. In contrast, skin-surface-to-artery distance or longitudinal axis deviation seemed to have no impact on cannulation success. 

Our results are in line with previous reports. Jung Oh et al. report reduced radial artery cross-sectional area as an independent predictor of first-attempt failed catheterization in children even when ultrasound was used [15]. Measures that may increase that diameter and cross-sectional area such as a median nerve block performed prior to radial artery cannulation will help facilitate the puncture [16]. 

In accordance with our data, the results from Kotowycz et al. revealed that lower patient height, weight, BMI and BSA, together with other physiognomical parameters such as wrist circumference or shoe size, predict reduced radial artery size in patients undergoing cardiac catheterization [17]. Consequently, difficulties encountered during radial cannulation leading to conversion into femoral access were similarly shown to be associated with reduced patient height and body surface area [18,19]. This is somewhat surprising since radial catheterization is usually thought to be particularly challenging in obese patients [20]. However, it was demonstrated that radial artery diameter is increased in obese patients compared with lean subjects, which possibly explains our finding that difficult cannulation was associated with reduced body mass index [21]. Interestingly, in contrast with our study, results from other studies revealed no association between clinical parameters such as BMI and radial artery diameter [22]. Nevertheless, they similarly stressed the significance of ultrasound in improving radial artery cannulation success. 

In our study, plaques and stenoses were equally distributed between the two groups, suggesting that radial artery quality has no impact on catheterization success. In fact, as revealed by a large study by Dehghani et al. involving more than 2000 patients, neither plaques nor stenoses were independently associated with failing transradial cannulation for percutaneous coronary intervention [23]. However, due to the small sample size in our study, an effect may not be excluded with certainty. A recent study by Achim et al. revealed a further interesting aspect, demonstrating that radial artery calcification is correlated with coronary calcification and plaque burden requiring revascularization [24]. This suggests that radial ultrasound may also be useful for preoperatively identifying patients with significant coronary atherosclerosis, underlining the role of the anesthesiologist in screening for relevant comorbidities.

Our study has some limitations, including a small sample size that potentially resulted in underpowered conclusions. Only orthopedic and cardiac surgery patients were evaluated, potentially limiting the application of our results to other surgical patient populations. Last, although performed uniformly in all patients by the same experienced operators, arterial cannulation was not strictly standardized but was left to the discretion of the anesthetist taking care of the patient. Since the classical forearm proximal access was used in all cases, our results cannot be transferred to other approaches such as the distal radial artery access performed at the level of the snuffbox, which has recently been proven to be non-inferior compared with the conventional proximal radial access [6,7].

## 5. Conclusions

In summary, the results of our observational study revealed that radial artery catheterization performed using pulse palpation may be difficult, especially in adult patients with lower body weight and height, due to their reduced arterial diameters. Initial ultrasound use in these patients may reduce first-attempt failure, preventing procedural delays and patient discomfort.

## Figures and Tables

**Table 1:** Results of preoperative ultrasound assessment of patients in the whole cohort as well as patients in group 1 (uncomplicated catheterization) and group 2 (difficult catheterization).

	Whole Cohort	Group 1	Group 2	
	*n* = 41	*n* = 25	*n* = 16	*p*-Value
**Basic characteristics:**				
Weight (kg)	82 (73–90)	83 (73–95)	75 (65–85)	**0.046**
Height (cm)	174 (167–181)	178 (170–182)	169 (162–178)	**0.011**
Body mass index	26.8 (23.8–29.2)	27.7 (24.2–29.1)	25.9 (22.7–29.3)	0.521
Body surface area (m^2^)	1.96 (1.80–2.12)	2.04 (1.83–2.17)	1.90 (1.67–1.96)	**0.011**
Heart rate (bpm)	68 (60–79)	68 (60–81)	66 (60–79)	0.796
Blood pressure sys (mmHg)	125 (110–136)	130 (110–141)	125 (110–134)	0.436
Blood pressure dia (mmHg)	75 (70– 82)	75 (70–85)	75 (70–80)	0.963
Previous arterial puncture (*n*)	31 (76%)	19 (76%)	12 (75%)	0.999
**Ultrasound characteristics:**				
Dist. int. diameter SA (mm)	2.60 (2.32–2.95)	2.64 (2.38–2.98)	2.41 (1.99–2.60)	**0.039**
Prox. int. diameter SA (mm)	2.61 (2.25–2.91)	2.64 (2.50–3.04)	2.41 (2.04–2.85)	0.058
Dist. int. diameter LA (mm)	2.48 (2.14–2.81)	2.60 (2.37–2.91)	2.32 (1.96–2.58)	**0.018**
Prox. int. diameter LA (mm)	2.57 (2.25–2.93)	2.63 (2.48–2.95)	2.30 (1.98–2.76)	**0.024**
Long axis deviation SA (mm)	4.66 (3.23–6.72)	5.24 (3.15–7.44)	4.31 (3.27–6.13)	0.594
Long axis deviation LA (mm)	1.00 (1.00–1.00)	1.00 (1.00–1.00)	1.00 (1.00–1.00)	0.999
Dist. distance skin–artery (mm)	6.43 (5.32–7.21)	6.65 (5.69–8.42)	6.16 (4.42–6.81)	0.084
Prox. distance skin–artery (mm)	7.01 (4.87–8.25)	7.01 (4.70–8.47)	6.96 (4.86–8.29)	0.911
Vmax (cm/s)	80.70 (61.95–99.55)	86.35 (64.53–141.00)	79.15 (55.65–95.83)	0.349
Stenoses, plaques (*n*)	14 (34%)	9 (36%)	5 (31%)	0.999

Data are given as median values with 25th and 75th percentiles or as absolute numbers with percentage values and were compared using the Mann–Whitney test or Fisher’s exact test, respectively. *p* values refer to the results of intergroup comparisons (group 1 vs. 2). Significant differences are given in bold values. SA = short axis scan, LA = long axis scan.

**Table 2:** Results of radial artery cannulation observations in the whole cohort as well as in group 1 (uncomplicated catheterization) and group 2 (difficult catheterization).

	Whole Cohort	Group 1	Group 2	
	*n* = 41	*n* = 25	*n* = 16	*p*-Value
Heart rate (bpm)	70 (61–80)	70 (59–80)	70 (63–80)	0.478
Blood pressure sys (mmHg)	141 (125–157)	144 (121–158)	138 (127–156)	0.706
Blood pressure dia (mmHg)	71 (62–83)	73 (61–87)	68 (63–78)	0.520
Pulsatility index	0.80 (0.50–1.80)	0.78 (0.50–1.29)	0.80 (0.50–2.70)	0.536
Number of attempts needed for successful catheter placement (*n*)	1 (1–2)	1 (1–1)	2 (2–3)	**0.0001**
Time needed for successful catheter placement (s)	77 (47–179)	53 (38–77)	181 (155–286)	**0.0001**
Secondary use of ultrasound (*n*)	8 (20%)	0 (0%)	8 (50%)	**0.0001**

Data are given as median values with 25th and 75th percentiles or as absolute numbers with percentage values and were compared using the Mann–Whitney test or Fisher’s exact test, respectively. *p*-values refer to the results of intergroup comparisons (group 1 vs. 2). Significant differences are given in bold values.

## Data Availability

The datasets used and/or analyzed during the current study are available from the corresponding author on reasonable request.
